# Serous Effusions Diagnostic Accuracy for Hematopoietic Malignancies: A Cyto-Histological Correlation

**DOI:** 10.3389/fmed.2020.615080

**Published:** 2020-12-03

**Authors:** Jinnan Li, Sha Zhao, Wenyan Zhang, Yong Jiang, Xianglan Zhu, Xueqin Den, Weiping Liu, Xueying Su

**Affiliations:** Department of Pathology, West China Hospital of Sichuan University, Chengdu, China

**Keywords:** hematopoietic malignancies, serous effusions, ancillary studies, cytology, cyto-histological correlation

## Abstract

**Background:** The aim of this study was to establish the liability of cytological diagnostic and, along with ancillary techniques, to sub-classify hematopoietic malignancies in serous effusions.

**Methods:** We retrospectively reviewed the serous effusions of hematopoietic malignancies over an 11-year period, along with ancillary studies, clinical and histological data. We compared cytological along with histological diagnosis to evaluate the value of cytology itself. Furthermore, the discrepant cases were reviewed.

**Results:** In this study, a total of 242 cases were identified as hematopoietic malignancies. Ancillary technologies were performed: in 24 cases FCM, 242 cases ICC, 35 cases ISH, 81 cases PCR and 10 cases FISH. Cyto-histological correlation was available for 122 cases. The subtyping of hematopoietic malignancies was achieved using cytological material in 65/122 cases (53.3%). Of the 65 cases, T-Acute lymphoblastic leukemia/lymphoma (22.1%) was the leading subtype, followed by Burkitt lymphoma (5.7%), plasmacytoma (5.7%). Cyto-histological correlation showed a 100% concordant rate of diagnosis for hematopoietic malignancies and a high degree of agreement on sub-classification (51.6%). In this regard, T-acute lymphoblastic leukemia/lymphoma, plasmacytoma, extranodal NK/T-cell lymphoma, nasal type, anaplastic large cell lymphoma, myeloid sarcoma, and follicular lymphoma showed the highest degree of agreement (100%). The sub-classification on cytology was achieved in 53 out of the remaining 120 cases without histological diagnosis (44.2%). T-acute lymphoblastic leukemia/lymphoma (20.8%) was again the most frequently encountered subtype, followed by plasmacytoma (5.8%) and Burkitt lymphoma (4.2%).

**Conclusions:** This large series study provided evidence that combining cytology and ancillary studies enabled the accurate serous effusions cytological diagnoses and subsequent sub-classification for the described malignancies.

## Introduction

Hematopoietic malignancies (HM) are common causes of malignant serous effusions (SE), which suggest advanced stage of disease and poor prognosis (1, 2). In malignant SE caused by HM, cytology remains the first-line, cost effective and rapid diagnostic method. Some previous studies evaluated the diagnostic value of SE cytology for HM, and a few of them analyzed the sub-classification (3–6). However, no studies involved cyto-histological correlation with WHO classification criteria. The objective of the current study is to investigate the diagnostic value of SE cytology for HM, by doing a cyto-histological correlation. Furthermore, the reasons of discrepancy are analyzed. Our experience of SE cytological diagnosis of an HM is also summarized. The main finding of this study, includes a proposal of a further appropriate approach for accurate diagnosis of HM, in SE, and proves the value of cytology for the HM described, as well as their sub-classification.

## Materials and Methods

### Study Cases

Archives of the pathology department of West China Hospital of Sichuan University, from 2008 to 2019, were researched for HM with SE. The diagnoses were in accordance with WHO classification of Tumors of Haematopoietic and Lymphoid Tissue (2008/2017) criteria (7, 8).

### Flow Chart of Cytological Diagnosis

**Figure 1:**
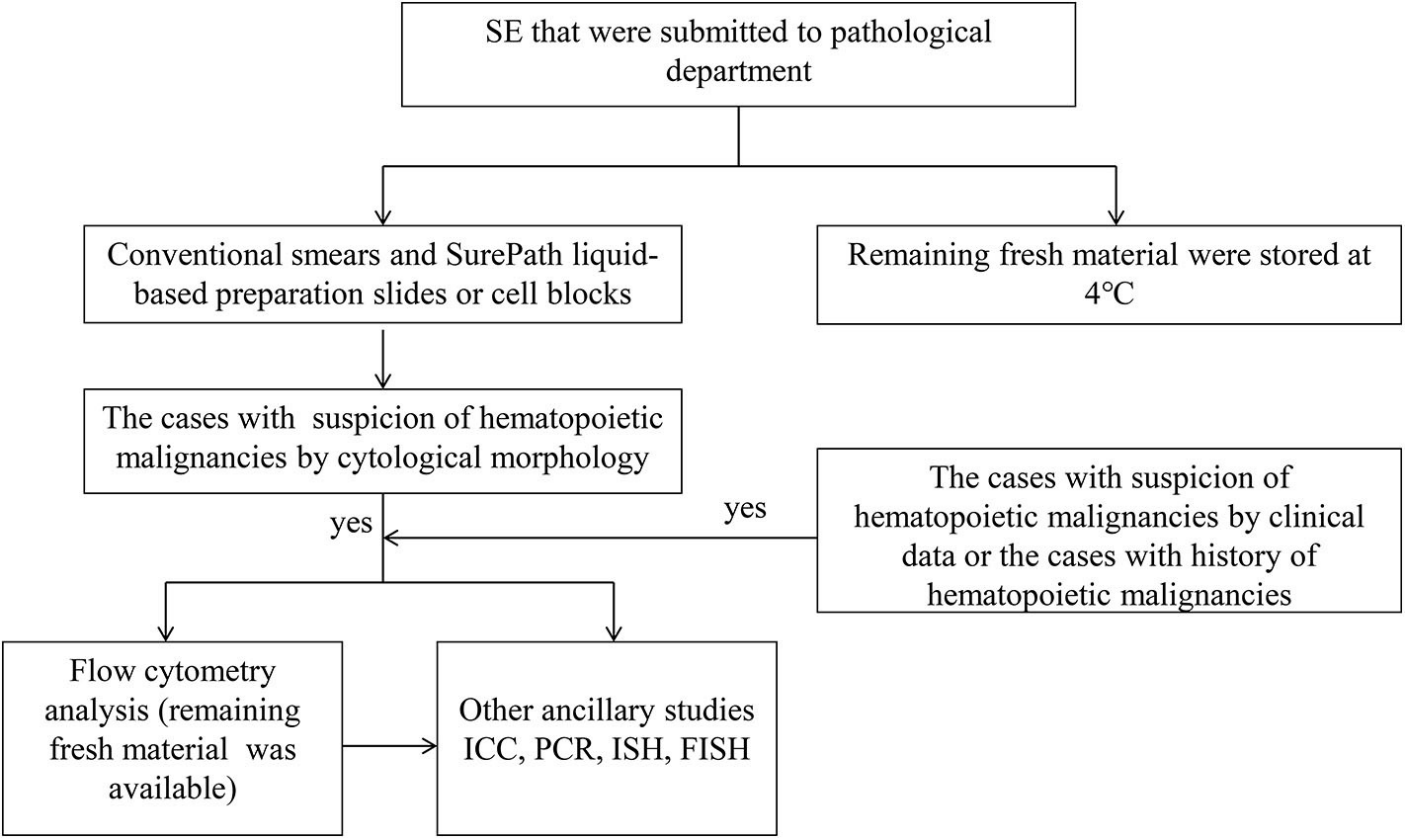
Diagnostic approach of HM in SE recommended. SE indicates serous effusion; ICC, immunohistochemistry; PCR, polymerase chain reaction; ISH, *in situ* hybridization; FISH, fluorescence *in situ* hybridization.

The conventional smears and SurePath, liquid-based preparation slides (BD, Franklin Lakes, NJ) or cell blocks, were prepared for all cases. Papanicolaou staining was performed on alcohol-fixed smears and liquid-based preparation slides. Diff-Quick staining was performed on air-dry slides in some cases. Haematoxylin and eosin stain (HE) staining was performed on cell block sections. Paraffin-embedded (FFPE) cell blocks or formalin-fixed liquid-based preparation slides were used for ancillary studies. The remaining fresh material was stored at 4°C. The flowchart was summarized in Figure 1. Cytomorphological assessment of all samples was performed within 24 h. An immediate flow cytometry (FCM) analysis was performed on SE submitted with either clinical or cytomorphological suspicion of HM. Abnormal cases indentified by FCM were submitted for further immunocytochemistry (ICC) and/or molecular assays.

### Immunophenotyping

FCM was performed by six-color immunofluorescent staining. The initial evaluation of the samples was based on a screening panel of three tubes with the appropriate combination of the following commercially available fluorescence-labeled antibodies: CD2, CD3, CD4, CD5, CD7, CD8, CD10, CD19, CD20, CD30, CD38, CD45, CD56, kappa light chain, and lambda light chain. More specific markers were applied in cases with abnormal findings. Data acquisition and analysis were performed using CellQuest software (Becton Dickinson).

ICC was performed on either SurePath preparation slides or cell block sections using the Envision method. The panel of specific markers depended on cytomorphology and/or result of FCM. The following antibodies were used: CD3ε, CD5, CD20, CD45RO, CD10, CD56, CD30, CD79a, CD99, CD138, BCL2, BCL6, MUM1, MYC, SOX11, ALK, GZM-B, TIA-1, TdT, CCND1, MPO, PC, and Ki-67.

### *In situ* Hybridization (ISH) for Epstein-Barr Virus (EBV) Encoded RNA

ISH was carried out with a fluorescein-labeled oligonucleotide probe complementary for 2 EBV-encoded small RNAs, EBER-1 and EBER-2 (EBER1/2) (Dako-Y520001). Rabbit anti-FITC antibody conjugated with alkaline phosphatase (AP) (Dako, Denmark) was used to combine with the probe, while NBT/BCIP was used as a substrate. The hybridizing signal located in the cell nucleus.

### PCR Assays

Genomic DNA, from FFPE cell blocks, was extracted by phenol-chloroform procedures. PCR analysis of B-cell and T-cell clonality by following the previous reported protocol (9).

### Fluorescence *In situ* Hybridization (FISH) Analysis

FISH analysis was carried out on the cell block sections, using probes specific for rearrangement detection. The following probes were used: Vysis LSI *BCL6* dual-color break-apart, Vysis LSI *BCL2* dual-color break-apart, Vysis LSI *MYC* dual-color break-apart, Vysis LSI *IGH/BCL2* dual-color and dual fusion probes. The hybridization protocol and the scoring criteria used for the assays have been published previously (10).

## Results

### Clinical Manifestations

A total of 188 pleural fluids, 49 ascites and 5 pericardial fluids from 242 patients (157 males, 85 females) were diagnosed as HM. The clinicopathologic data was listed in Table 1. The mean patient age was 47.2 years-old (range 2–91 years). The SE samples represented the initial source of diagnosis in 167/242 patients (69.0%). The histological diagnoses were available for 122 cases (50.4%). For this group, the SE samples represented the initial source of diagnosis in 47/122 cases (38.5%). There were 36 cases (36/122, 29.5%) with concurrent histopathological biopsy. The remaining 39 patients (39/122, 32.0%) had previous histological diagnoses of HM.

**Table 1:** Characteristics of patients and samples.

**Patient characteristics**	**No. of patients (%)**
**Sex**	
Male	157 (64.9%)
Female	85 (35.1%)
Age, year	2–91
Mean	47.2
**Previous history of hematopoietic malignancies**	
Yes	39 (16.1%)
No	53 (83.9%)
**Location of effusions**	
Pleural	188 (77.7%)
Peritoneal	49 (20.2%)
Pericardial	5 (2.1%)
**Cell block or SurePath**	
Cell block	211 (87.2%)
SurePath	31 (12.8%)

### Evaluation of Cytomorphology

**Figure 2:**
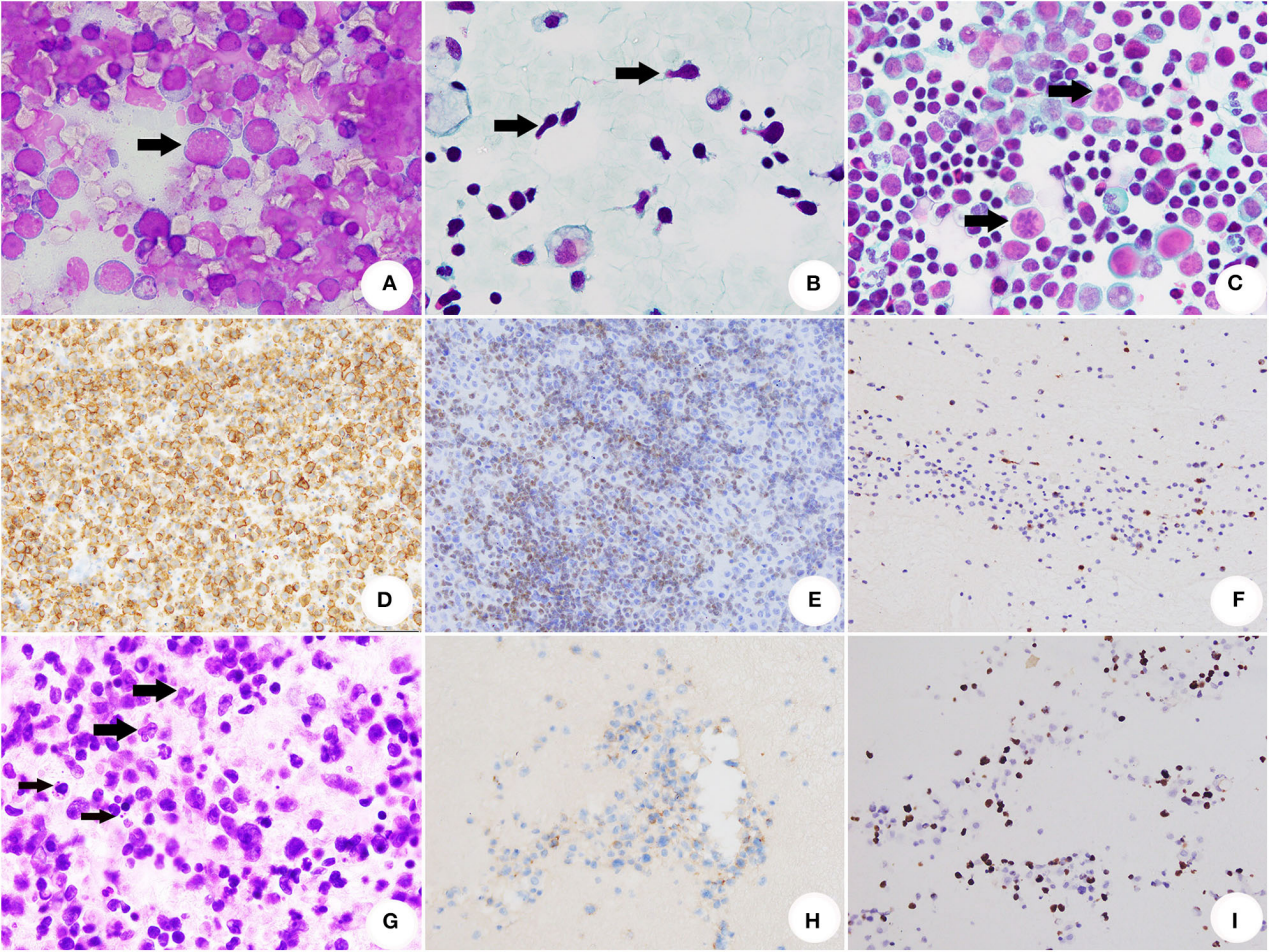
Cytomorhpology, ICC and ISH of SE caused by HM. **(A)** The conventional smear of BL showed non-cohesive atypical lymphocytes with cytoplasmic/nuclear (DiffQuik® stain, original magnification × 1,000). **(B)** The conventional smear of T-ALL/LBL. The arrows indicated several lymphoblasts with hand mirror-shaped morphology (Papanicolaou stain, original magnification × 1,000). **(C)** The conventional smear of AITL. The arrows indicated abnormal mitosis (Papanicolaou stain, original magnification × 1,000). **(D)** The neoplastic cells of BL were positive for CD20 (ICC stain, original magnification × 400). **(E)** T-ALL/LBL was positive for TDT (ICC stain, original magnification × 400). **(F)** AITL was illustrated by positivity of CXCL13 (ICC stain, original magnification × 400). **(G)** Section of cell block for ENKTCL. The thick arrows indicated medium-sized and large-sized atypical presented irregularly folded nuclei with the granular chromatin. Nucleoli were generally inconspicuous or small. The thin arrows indicated apoptosis (HE, original magnification × 1,000). **(H)** Neoplastic cells of ENKTCL were positive for CD56 (immunocytochemistry stain, original magnification × 400). **(I)** EBV infection of ENKTCL was demonstrated by ISH for EBER (Epstein-Barr virus-ISH, original magnification × 400).

**Figure 3:**
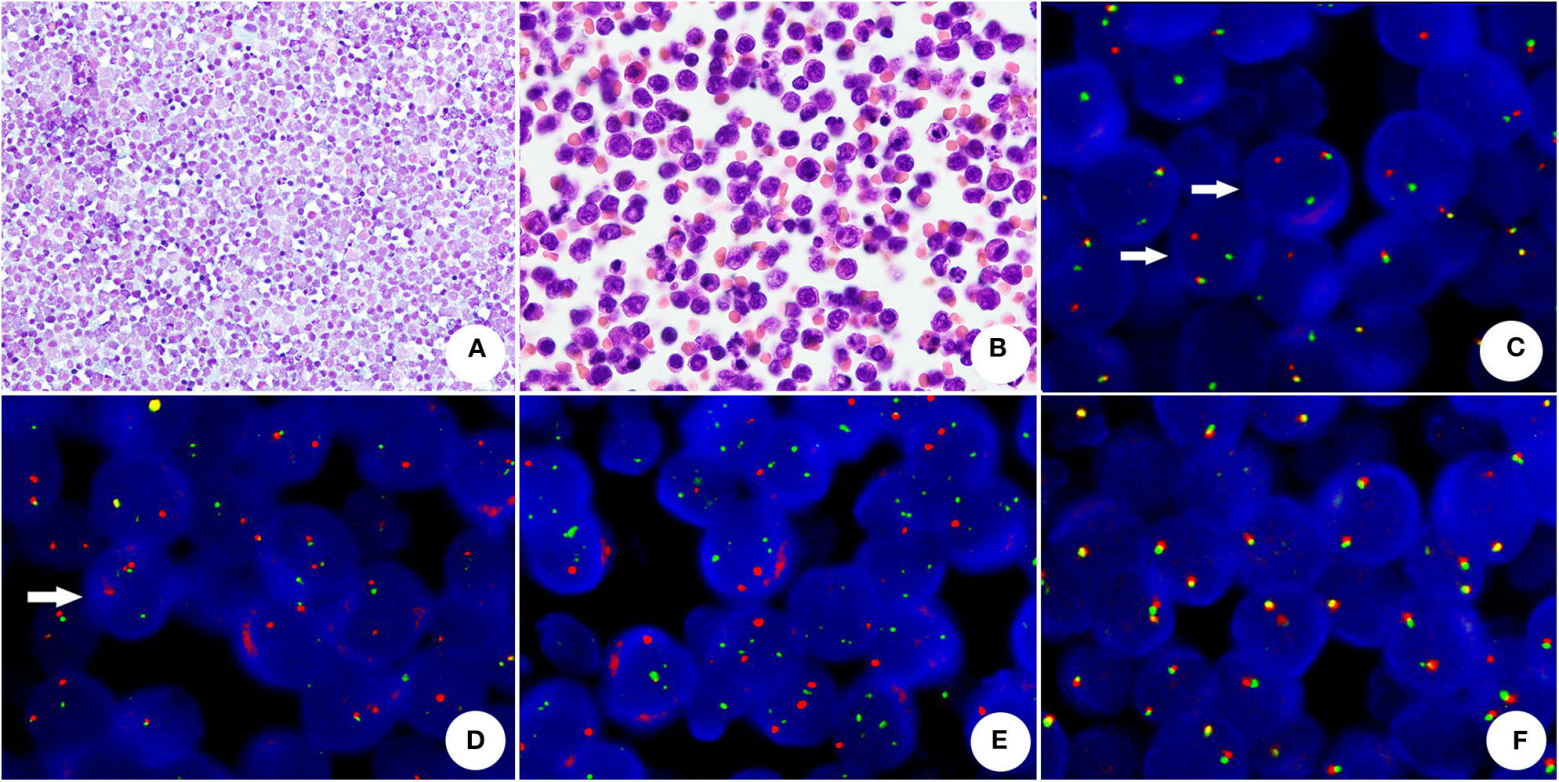
The cytomorphology and FISH assay of HGBL with *MYC* and *BCL2* rearrangements **(A)** The conventional smear presented uniformed and non-cohesive atypical lymphocytes with numerous cytoplasmic/nuclear vacuoles (Papanicolaou stain, original magnification × 400). **(B)** The cell block section showed medium atypical lymphocytes with round or oval nuclei, chromatin and micronucleoli (HE, original magnification × 1,000). **(C)** *BCL2* rearrangement-positive tumor cells, showing one split red and green signals (arrow) and one yellow fusion signal (dual-color, break-apart probe). **(D)** *MYC* rearrangement was demonstrated by split red-green signals (arrow) and a normal fusion signal in a neoplastic cell (dual-color, break-apart probe). **(E)** Absence of *MYC/IGH* rearrangement (dual-color, dual-fusion probe), neoplastic cells did not reveal abnormal fusion signals. **(F)** The neoplastic cells without *BCL6* rearrangement showed normal fusion signals (dual-color, break-apart probe).

There were cytomorphological features which provided clues for the diagnosis of HM: medium to large-sized atypical lymphocytes (Figure 2A), unexplainable immature lymphocytes (hand mirror-shaped blasts) (Figure 2B), irregular lymphocytes (Figure 2G), prominent mitotic figures and unexplainable apoptosis (Figures 2C,G), uniformed and diffused lymphocytes (Figures 3A,B).

## Ancillary Studies

### FCM and ICC

FCM was performed in 24 samples and identified 15 B-cell lymphomas, 7 T-cell lymphomas and 2 NK/T-cell lymphomas. ICC (Figures 2D–F,H) was achieved in all the cases and revealed 156 B-cell lymphomas (64.5%), 74 T-cell lymphomas (30.6%), 6 NK/T-cell lymphomas (2.5%), 3 myeloid neoplasm (1.2%) and 3 precursor lymphoid neoplasm (1.2%) (Cell lineage was unclassified).

### ISH for EBV Encoded RNA

Thirty-five samples were submitted for EBER1/2 analysis (Figure 2I), and positive staining was demonstrated in 8/35 cases (22.9%).

### PCR Assays

PCR analysis for B-cell and/or T-cell monoclonality was performed in 81 samples. A total of 72 positive cases, including 49 cases with B-cell monoclonality, 17 cases with T-cell monoclonality and 6 cases with both B and T-cell monoclonality, were identified. The remaining 9 negative cases, included: 2 without B-cell monoclonality, 5 without T-cell monoclonality and 2 without both B-cell and T-cell monoclonality.

### FISH Analysis

FISH analysis was performed in 10 cases. There were 8 positive cases: 4 with both *IGH/MYC* and *MYC* rearrangements, 1 with isolated *MYC* rearrangement, 1 with isolated *IGH/MYC* rearrangement and 2 with both *MYC* and *BCL2* rearrangements (Figures 3C–F). For the 2 remaining cases no rearrangements were found.

## Cytological Diagnoses

### Group of Patients With Histological Correlation (122 Cases)

The cytological diagnoses of 78 B-cell lymphomas, 35 T-cell lymphomas, 6 NK/T-cell lymphomas, 2 myeloid neoplasms and 1 ALL/LBL, were established according to cytomorphology and ancillary studies. The specific subtyping of HM was achieved in 65/122 cases (53.3%). Of these 65 cases, T-acute lymphoblastic leukemia/lymphomas (T-ALL/LBL) was the leading subtype (27 cases), followed by 7 Burkitt lymphomas (BL), 7 plasmacytomas, 6 extranodal NK/T-cell lymphomas, nasal type (ENKTCL), 3 mantle cell lymphomas (MCL) and 3 anaplastic large cell lymphomas (ALCL). The cyto-histological correlation showed a 100% overall concordance rate of diagnosis of HM and a high agreement rate of sub-classification (51.6%). In this regard, T-ALL/LBL, plasmacytoma, ENKTCL, ALCL, myeloid sarcoma (MS) and follicular lymphoma (FL) showed the highest degree of agreement (100%). According to the cyto-histological correlation, the agreement rate of BL, B-ALL/LBL, angioimmunoblastic T cell lymphoma (AITL), MCL, Indolent B-cell lymphoma and ALL/LBL was 77.8, 66.6, 66.6, 60.0, 50.0, and 33.3%, respectively. Limitation of cytology, inadequate ancillary studies and false negative of ICC were the main reasons for discrepancy. Due to limitation of cytology and/or inadequate ancillary studies, the sub-classification of cytological diagnoses was failed in splenic marginal zone lymphoma (SMZL), chronic lymphocytic leukemia/small lymphocytic lymphoma (CLL/SLL), diffuse large B-cell lymphoma (DLBCL), lymphoplasmacytic lymphoma (LPL), mucosa-associated lymphatic tissue lymphoma (MALT lymphoma), Primary cutaneous gamma delta T-cell lymphoma (PCGD-TCL), primary mediastinal large B-cell lymphoma (PMLBL) and High-grade B-cell lymphoma (HGBL), NOS. From the point of histological diagnoses, the most common type was DLBCL (38 cases, 31.4%), followed by T-ALL/LBL (25 cases, 20.5%), BL (8 cases, 6.6%), plasmacytoma (7 cases, 5.7%), ENKTCL (6 cases, 4.9%) and MCL (5 cases, 4.1%). However, most of DLBCL were diagnosed as large B-cell lymphomas (LBCL) by cytology. The cytological and histological diagnoses, as well as diagnostic coincidence rates and reasons for discrepancy were summarized in Table 2.

**Table 2:** The cyto-histological correlation of patients with histological correlation.

**Histological diagnosis**	**No. of cases**	**Cytological diagnosis**	**Coincidence rate**	**Reason for discrepancy**
BL	9	7 BL 2 B-cell NHL	77.8%	Inadequate ancillary studies
B-ALL/LBL	3	2 B-ALL/LBL 1 ALL/LBL	66.6%	False negative of ICC
MZL	5	3 MZL 2 Indolent B-cell lymphoma	60.0%	Inadequate ancillary studies
SMZL	1	1 B-cell NHL	0	Limitation of cytology
CLL/SLL	2	2 Indolent B-cell lymphoma	0	Inadequate ancillary studies
DLBCL	38	30 LBCL 8 B-cell NHL	0	Limitation of cytology
Plasmacytoma	7	7 Plasmacytoma	100%	/
MCL	5	3 MCL 2 B-cell NHL	60.0%	Inadequate ancillary studies
FL	1	1 FL	100%	/
LPL	1	1 B-cell NHL	0	Limitation of cytology
MALT lymphoma	1	1 B-cell NHL	0	Inadequate ancillary studies
HGBL with *MYC* and *BCL2* rearrangement	1	1 HGBL with MYC and BCL2 rearrangement	100%	/
HGBL, NOS	1	1 B-cell NHL	0	Inadequate ancillary studies
Indolent B-cell lymphoma	2	1 Indolent B-cell lymphoma 1 B-cell NHL	50%	Inadequate ancillary studies
ENKTCL	6	6 ENKTCL	100%	/
T-ALL/LBL	25	25 T-ALL/LBL	100%	/
ALCL	3	3 ALCL	100%	/
AITL	3	2 AITL 1 TCL	66.6%	Inadequate ancillary studies
PCGD-TCL	1	1 TCL	0	Limitation of cytology
PTCL, NOS	1	1 PTCL, NOS	100%	/
PMLBL	1	1 LBCL	0	Limitation of cytology
Myeloid sarcoma	2	2 Myeloid sarcoma	100%	/
ALL/LBL	3	2 T-ALL/LBL 1 ALL/LBL	33.3%	/

*BL, Burkitt lymphoma; ALL/LBL, lymphoblastic leukemia/lymphoma; MZL, marginal zone B-cell lymphoma; SMZL, splenic marginal zone lymphoma; CLL/SLL, chronic lymphocytic leukemia/small lymphocytic lymphoma; DLBCL, Diffuse large B-cell lymphoma; MCL, Mantle cell lymphoma; FL, Follicular lymphoma; LPL, Lymphoplasmacytic lymphoma; MALT lymphoma, mucosa-associated lymphatic tissue lymphoma; HGBL, High-grade B-cell lymphoma with MYC and BCL2 rearrangement; ENKTCL, Extranodal NK/T-cell lymphoma, nasal type; ALCL, Anaplastic large cell lymphoma; AITL, Angioimmunoblastic T cell lymphoma; PCGD-TCL, Primary cutaneous gamma delta T-cell lymphoma; PTCL, NOS, Peripheral T-cell lymphoma, not otherwise specified; PMLBL, primary mediastinal large B-cell lymphoma*.

### Group of Patients Without Histological Correlation (120 Cases)

The diagnostic of 78 B-cell lymphomas, 39 T-cell lymphomas, 1 myeloid neoplasm and 2 ALL/LBLs, was established according to cytomorphology and ancillary studies. The specific subtyping of HM was achieved in 53/120 (44.2%) cases. Of these 53 cases, T-ALL/LBL was also the leading subtype (25 cases), followed by plasmacytomas (7 cases), BL (5 cases), B-ALL/LBL (5 cases) and ALCL (5 cases). These cytological diagnoses were summarized in Table 3.

**Table 3:** The cytological diagnoses of patients without histological correlation.

**Cytological diagnosis**	**No. of cases (%)**
B-cell lymphoma	78 (65.0%)
B- cell NHL	28 (23.3%)
LBL	26 (21.7%)
Indolent B-cell lymphoma	3 (2.5%)
Plasmacytoma	7 (5.8%)
BL	5 (4.2%)
B-ALL/LBL	5 (4.2%)
PEL	2 (1.7%)
MCL	1 (0.8%)
HGBL with *MYC* and *BCL2* rearrangement	1 (0.8%)
T-cell lymphoma	39 (32.5%)
Mature T-cell lymphoma	8 (6.7%)
T-ALL/LBL	25 (20.8%)
ALCL	5 (4.2%)
PTCL, NOS	1 (0.8%)
Myeloid sarcoma	1 (0.8%)
ALL/LBL	2 (1.7%)

*NHL, Non-Hodgkin's lymphoma; LBL, Large B-cell lymphoma; BL, Burkitt lymphoma; B-ALL/LBL, Lymphoblastic leukemia/lymphoma; PEL, Primary effusion lymphoma; MCL, Mantle cell lymphoma; HGBL, High-grade B-cell lymphoma; ALL/LBL, Lymphoblastic leukemia/lymphoma; ALCL, Anaplastic large cell lymphoma; PTCL, NOS, Peripheral T-cell lymphoma, not otherwise specified*.

## Discussion

The use of cytological evaluation of SE for diagnostic purposes of HM has been taken with controversy, particularly in the setting of initial diagnostic and sub-classification. The current study assessed cytology combined with ancillary studies as the first line diagnostic tool for SE, in the context of HM, by having a cyto-histological correlation. Nowadays, this report demonstrated the largest study of cytological diagnosis of HM in SE.

In our practices, ancillary studies including FCM, ICC, ISH, PCR and FISH, as adjunct to cytomorphologic evaluation contributed to subtyping of the HM in 118/242 cases (48.8%). The subtyping rate was 33.0% in a report from Canada (3). The different diagnostic process and the differences in the distribution of HM between Asian and Western populations were likely the main reasons for the discrepancies. Previous studies suggested that distribution of HM subtypes, using SE, differed strikingly by geographic variations (3, 5, 6). T-ALL/LBL was the most common type and account for 21.5% of HM in our study. A similar result was reported in an Indian study, T-ALL/LBL was also the most common subtype (36.9%) (6). However, the ratio of T-ALL/LBL in an European report was only 3.1% (3). In our study, the 3 most frequent subtypes, either with or without histological diagnosis were similar. T-ALL/LBL was the most common subtype, followed by plasmacytoma and BL. ENKTCL accounted for 4.1% in the group of patients with histological diagnosis, and it was not to be diagnosed in the group of patients without histological correlation. There were three possible reasons for this: in the first place, ENKTCL rarely present SE as initial symptom; secondly, inadequate ancillary studies leaded to the failure of sub-classification; finally, limitation of physician diagnostic experience also contributed to sub-classification failure. In another previous study from ours, 3/5 cases of ENKTCL were initially diagnosed by cytological examination (11). Considering all these aspects, the latter two reasons were likely to contribute to the differences. According to histological diagnosis in the group of patients with histological correlation, DLBCL (38/122, 31.1%) was the most common type. Even though there was no cytological diagnosis without histological correlation, LBCL was the most frequent entity found. Limitations of cytology itself may contribute for this discrepancy.

In addition, T-ALL/LBL, BL, plasmacytoma, and ENKTCL also accounted in the top five subtypes of histological diagnoses, which were similar to cytological diagnoses. Fifty-nine cases had discrepancies of sub-classification between cytological and histological diagnoses. Upon review of these discordant cases, inadequate ancillary studies, false negative results, limitation of physician diagnostic experience and limitations of cytological diagnosis were likely the main reasons for these discrepancies. In this group, cell blocks were available in 91/122 cases (74.6%), and in the remaining 31 cases, SurePath liquid-based preparation was performed, after making traditional smears. Limited ancillary studies in these 31 cases leaded to the difficulty of sub-classification. In addition, false negative results, especially for immunophenotype, contributed to challenge of cytological diagnoses (9, 10, 12). Cytology, coupled with FMC, highlighted it with an important role in the diagnosis and sub-classification of HM (12–19). In our practice, cytology combined with FCM also could contribute to sub-classification of HM in SE samples. In our opinion, the value of FCM had more importance for mature T-cell lymphoma diagnosis. Most mature T-cell lymphomas mixing with various reactive cells including B lymphocytes, eosinophils, histocytes, and mesothelial cells leaded to the challenge in sub-classification on cytology. FCM had a high sensitivity to lead to the detection of complex lesion. Two cases of AITL and 2 cases of peripheral T-cell lymphoma, not otherwise specified (PTCL, NOS), were successfully diagnosed by using cytology along with FCM, ICC and PCR.

Some types of HM which have specific cytomorphologic features, immunophenotyping or molecular genetic changes can be diagnosed by cytology. In the first approach to the patient, cytomorphological features are important diagnostic clues for most of lymphomas. From our observations, there are some characteristics that might point out a specific diagnosis. For example: nuclear cleft and “hand mirror-shaped like” cells are features of ALL/LBL; cytoplasmic and/or nuclear vacuoles are most frequently present in BL; ALCL reveals classic hallmark cells. It is required, subsequently, a panel of immune markers for sub-classification, like: TdT, CD34, Cd117, and CD99 for ALL/LBL (9); CD5, SOX11, and CCND1 for MCL (20); CD3, CD56, TIA-1, GZM-B, and EBER-ISH for ENKTCL (11). Thirdly, some lymphomas reveal hallmark cytogenetic abnormalities, such as: *t*(8, 14) (q24;q32), or its variants, was demonstrated in most cases of BL (8, 21); *MYC* rearrangement accompanied with *BCL2* or *BCL6* gene rearrangement was detected in “double hit” lymphomas (DHL). Finally, clinical manifestations and laboratory parameters also play a great role in the cytological diagnosis. Some specific subtypes of HM with special clinical manifestations, such as breast implant-associated anaplastic large cell lymphoma (BI-ALCL) occurring on peri-implant breast seroma and primary effusion lymphoma (PEL), were diagnosed better with cytology then with histology (22, 23).

In conclusion, our study is the largest series review of SE cyto-histological correlation for HM diagnosis. Our data supported the finding that SE cytology could be a reliable and accurate diagnostic tool for the diagnosis and sub-classification of HM. The clinical information, cytomorphology and appropriate ancillary studies equally contributed to an accurate diagnosis and sub-classification.

## Data Availability Statement

The original contributions presented in the study are included in the article/supplementary material, further inquiries can be directed to the corresponding author/s.

## Ethics Statement

The studies involving human participants were reviewed and approved by Ethics Committee On Biomedical Research, West China Hospital Of Sichuan University. The ethics committee waived the requirement of written informed consent for participation. Written informed consent was not obtained from the individual(s) for the publication of any potentially identifiable images or data included in this article.

## Author Contributions

JL conceived the idea, designed the study, and wrote the paper. SZ, WZ, and WL performed histological correlation. SZ, YJ, XZ, and XD performed ancillary studies. XS and WL supervised the research. All authors contributed to the article and approved the submitted version.

## Conflict of Interest

The authors declare that the research was conducted in the absence of any commercial or financial relationships that could be construed as a potential conflict of interest.
